# Adaptations in red blood cell indices and physical performance following bungy pump training in postmenopausal women

**DOI:** 10.3389/fphys.2026.1824312

**Published:** 2026-04-17

**Authors:** Yintao Niu, Monika Wiech, Guoping Qian, Sujie Mao, Zbigniew Ossowski

**Affiliations:** 1 Faculty of Physical Culture, Gdansk University of Physical Education and Sport, Gdansk, Poland; 2 School of Physical Education, Chizhou University, Chizhou, China; 3 Graduate Development Office, Harbin Sport University, Harbin, China

**Keywords:** bungy pump training, cardiorespiratory fitness, Nordic walking, physical performance, postmenopausal women, red blood cell indices

## Abstract

**Objective:**

The study examined the effects of a 12-week Bungy Pump Training (BPT) program on red blood cell (RBC) indices, physical performance, and anthropometric parameters in postmenopausal women (PW).

**Methods:**

Fifty-six PW were allocated to the BPT group (n = 30) and the Daily Activity (DA) group (n = 26). The BPT group participated in supervised 60-minute sessions three times per week for 12 weeks, while the DA group performed daily activities. RBC indices, physical performance (6-minute walk test, 6MWT), and anthropometric parameters were assessed before and after the intervention. Trial registration: Clinical Trials NCT06781541 on 17/01/2025.

**Results:**

A significant interaction was observed for mean corpuscular volume (p < 0.001), with an increase in the BPT group and a decrease in the DA group. Significant time effects were found for RBC, hemoglobin, hematocrit, and 6MWT (all p < 0.05). Following the intervention, the BPT group showed reductions in RBC and hemoglobin and an improvement in 6MWT, while the DA group also demonstrated an increase in 6MWT. A significant group effect indicated greater improvement in 6MWT (BPT: p = 0.018, d = 0.42; DA: p = 0.004, d = 0.35) in the BPT group compared with the DA group. A significant between-group difference in red cell distribution width–coefficient of variation change was observed; however, within-group changes were not significant. Additionally, reductions in waist circumference, waist-to-hip ratio, and arm circumference were observed in the BPT group.

**Conclusions:**

A 12-week BPT program was associated with changes in RBC indices and improvements in physical performance in PW. BPT was safe and well-tolerated, and may represent a feasible non-pharmacological strategy to support functional capacity and aspects of cardiovascular health in aging populations. Further studies are needed to confirm these findings.

## 1 Introduction

Cardiovascular diseases (CVDs) remain the leading cause of morbidity and mortality worldwide (Vaduganathan et al., 2022). Global cardiovascular mortality is projected to increase by 73.4% over the next 25 years, from 20.5 million deaths in 2025 to 35.6 million deaths in 2050 (Tsampasian and Bloomfield, 2024). With the rapid ageing of the global population, age has emerged as a major non-modifiable risk factor for CVDs (K et al., 2023), accompanied by progressive declines in physiological functions related to oxygen transport capacity, cardiovascular performance, and physical performance (Gifford and Collins, 2021; AlGhatrif et al., 2024). PW represent a unique physiological population in this context, as hormonal changes after menopause accelerate functional deterioration and elevate cardiovascular risk (Xia and Khalil, 2025). Understanding exercise-induced adaptations in physiological markers related to oxygen delivery capacity and physical performance is particularly important in this group.

Cardiorespiratory fitness (CRF) is a strong and consistent predictor of morbidity and mortality among adults, as it reflects the integrative capacity of the cardiovascular, respiratory, and muscular systems to deliver and utilize oxygen (Lang et al., 2024). In PW, CRF progressively declines due to age-related physiological changes, menopause-associated hormonal reductions, decreased muscle strength, and adverse shifts in fat distribution (Khalafi et al., 2023; Nunes et al., 2024).

Several physiological, physical performance, and anthropometric parameters—including red blood cell (RBC) indices, the 6-minute walk test (6MWT), and body circumference—are closely linked to cardiovascular health (Nebeck et al., 2012; Boreskie et al., 2020; Powell-Wiley et al., 2021; Jiang et al., 2024). These associations may present differently in PW due to complex hormonal and metabolic alterations following menopause (Xia and Khalil, 2025). Among these indicators, RBC indices are key physiological determinants of oxygen transport capacity, as hemoglobin within RBC enables efficient oxygen carriage and delivery throughout the circulation, thereby influencing systemic oxygen delivery and aerobic performance (Sitina et al., 2024). In addition, the red cell distribution width–coefficient of variation (RDW-CV) has emerged as an independent prognostic biomarker for CVDs (Haybar et al., 2019) and has been shown to correlate with CRF, particularly maximal oxygen uptake (VO_2_max) (Sobczak et al., 2023a).

Functional performance measures, such as the 6MWT, provide a validated assessment of submaximal aerobic capacity and serve as predictors of CRF, offering an indirect estimate of oxygen consumption during exercise (Mänttäri et al., 2018). Both CRF and skeletal muscle mass decline with advancing age, leading to reductions in muscle strength and physical function (Janssen, 2010). Anthropometric parameters, including waist circumference (WC) and waist-to-hip ratio (WHR), reflect visceral fat distribution (Gordito Soler et al., 2025), while reduced limb circumference may indicate sarcopenia, which is associated with impaired metabolism and elevated cardiovascular risk in older adults (Srikanthan and Karlamangla, 2014). Collectively, the assessment of these physiological and functional parameters is valuable for the early identification and management of metabolic and cardiovascular risk in PW.

Regular physical activity and structured exercise training are well-established strategies for improving CRF and cardiovascular health in PW (Ruiz-Rios and Maldonado-Martin, 2022; Sanchis-Gomar et al., 2022). However, the literature highlights gaps regarding the comparative effects of different exercise modalities on the overall cardiovascular health profile of this population. Sun et al. examined the effects of five training types—including continuous endurance training, interval training (INT), resistance training, aerobic combined with resistance training (combined training, CT), and hybrid-type training—on vascular function in PW, and identified CT and INT as the most effective modalities for improving vascular function (Sun et al., 2024). While vascular outcomes are frequently investigated, a more comprehensive evaluation incorporating hematological, physical performance, and anthropometric parameters may provide a broader and more physiologically relevant assessment of cardiovascular health in PW. Previous studies have demonstrated that CT improves CRF and reduces arterial stiffness in PW (Ferreira et al., 2024). In addition, aerobic exercise performed at moderate-to-vigorous or vigorous intensity has been shown to elicit greater improvements in VO_2_max and overall cardiovascular function in PW, with evidence from meta-analyses indicating that exercise type, intensity, and duration collectively influence CRF and muscular strength adaptations in this population (Khalafi et al., 2023). Accordingly, CT performed within this effective intensity range appears to be a particularly promising strategy for improving CRF and reducing cardiovascular risk in PW.

Nordic walking (NW) has emerged as an effective and accessible exercise modality for older adults. Recent evidence from systematic reviews and meta-analyses indicates that NW improves cardiovascular responses, physical performance and VO_2_max, and is associated with reductions in key cardiovascular risk factors, including blood pressure, lipid profiles, and adiposity (Dhamayanti et al., 2025; Liu and Kim, 2025). Similarly, walking-based interventions, including brisk walking, are associated with improved cardiovascular health and reduced cardiovascular disease risk (Paluch et al., 2023), with higher walking speed and greater physical activity levels linked to lower morbidity and mortality (Nurrobi et al., 2024). Together, these findings highlight the effectiveness of walking-based exercise modalities in improving cardiovascular health in older adults. Despite these well-documented benefits, most studies have primarily focused on cardiovascular, metabolic, and physical performance, while the effects of walking-based interventions on hematological parameters, particularly RBC indices, remain poorly understood.

Bungy Pump Training (BPT) is a form of CT that integrates aerobic and resistance components through the use of modified NW poles equipped with built-in resistance shock absorbers (Huta-Osiecka et al., 2022). By increasing external resistance during locomotion, BPT enables simultaneous stimulation of the cardiovascular and musculoskeletal systems (Wiacek et al., 2023). Wochna et al. demonstrated that BPT activates a greater number of muscle groups in PW compared with traditional NW (Wochna et al., 2022). Similarly, Ewa et al. reported improvements in physical performance following BPT in older adults; however, that study included both sexes and lacked a control group (Rodziewicz-Flis et al., 2023).

Previous studies have shown that both BPT and NW significantly increase VO_2_max in PW (Domaszewska et al., 2020; Sobczak et al., 2023a), suggesting favorable effects on CRF. However, evidence regarding their effects on physical performance, such as the 6MWT, remains limited. To date, only one study has reported significant reductions in RBC, hematocrit, and RDW-CV following twice-weekly BPT in PW (Sobczak et al., 2023a), and these findings warrant further confirmation. Based on recommendations from the American College of Sports Medicine (ACSM) and the American Heart Association, a training frequency of three sessions per week is generally considered optimal for older adults (Nelson et al., 2007), highlighting the need to further investigate the physiological and functional adaptations to BPT using training protocols consistent with current exercise guidelines. Therefore, BPT may represent a promising modality to simultaneously influence hematological and functional adaptations in PW; however, this has not yet been systematically investigated.

Given the growing popularity of BPT among older adults in Europe (Skórkowska-Telichowska et al., 2016), it is important to evaluate its effects on RBC indices and physical performance in PW. We hypothesized that BPT would lead to improvements in RBC indices and physical performance in PW following a 12-week intervention. Enhancing CRF through BPT may have potential clinical relevance for cardiovascular health; however, further evidence is needed. Therefore, the present study aimed to examine the effects of a 12-week BPT program (three sessions per week) on RBC indices and physical performance in PW.

## 2 Methods

### 2.1 Study design

**Figure 1:**
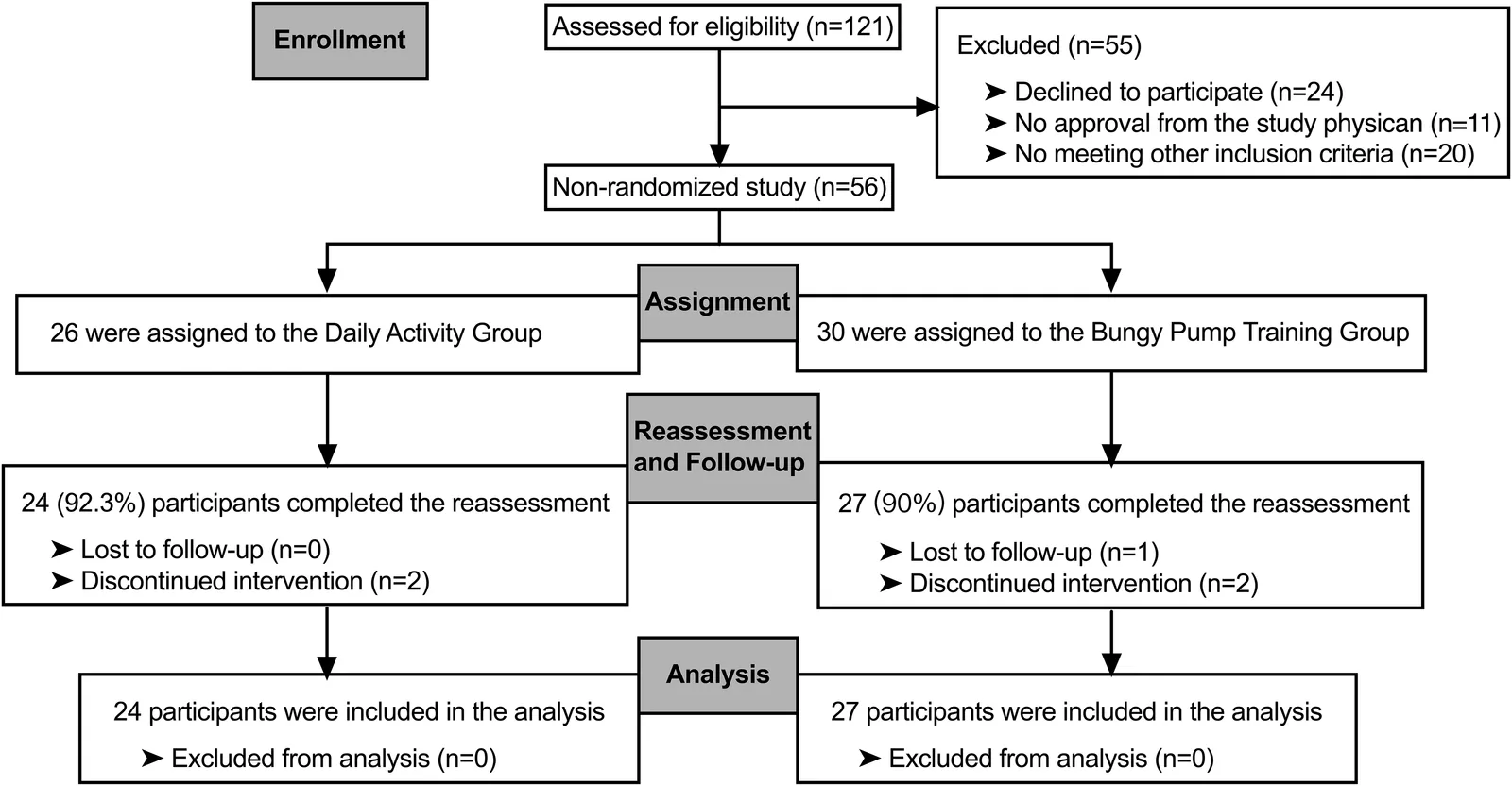
Study design and timeline of participant flow.

This study was registered at ClinicalTrials.gov (NCT06781541) which consisted of a non-randomized controlled trial with two parallel groups: the BPT group and the Daily Activity (DA) group. The trial was carried out in Gdańsk, Poland. Group allocation was determined using a geographic location–based quasi-randomization approach following baseline assessment. The study design and timeline are presented in **Figure 1**.

The study employed a pretest–posttest design with repeated measures to evaluate changes in outcomes, including RBC indices, physical performance, and anthropometric parameters, assessed at baseline and after completion of the intervention period. This design enabled the examination of within-subject changes over time as well as between-group differences. Participants assigned to the BPT group were supervised during all training sessions to ensure adherence and safety, whereas participants in the DA group were monitored for habitual physical activity over seven consecutive days. All participants were instructed to maintain their usual diet throughout the study period.

The study protocol was approved by the Bioethics Committee of the District Medical Chamber in Gdańsk (KB-5/22) and was conducted in accordance with the Declaration of Helsinki. All participants were fully informed about the study procedures and potential adverse events prior to enrolment and provided written informed consent.

### 2.2 Participants

PW were recruited through invitations distributed at the University of the Third Age in Gdansk and Sopot. Additional recruitment was conducted at local clinics, churches, and notice boards. All participants were enrolled on a voluntary basis.

Participant screening was conducted in two sequential phases. The initial phase involved administration of a self-administered questionnaire to obtain data on chronological age, menopausal status—defined as the absence of menstruation for ≥12 consecutive months—and general health status. Individuals who met the preliminary eligibility criteria subsequently underwent a comprehensive medical assessment to confirm eligibility for participation in the study. This assessment included a detailed review of comorbidities, current medications, and habitual physical activity levels.

Participants were eligible for inclusion if they met the following criteria: (1) postmenopausal status for at least 12 months; (2) age ≥60 years. Exclusion criteria were: (1) engagement in any structured or regular physical exercise program (e.g., aerobic training, resistance training, or NW), participation in more than 150–300 minutes of moderate-intensity aerobic activity per week (or an equivalent volume of vigorous-intensity activity), or prior exposure to BPT within the six months preceding enrollment; (2) uncontrolled hypertension, rheumatoid arthritis, or type 2 diabetes; (3) current or previous antibiotic and/or antifungal therapy within the past 4 weeks; (4) any medical contraindications to participation in physical exercise.

The sample size was estimated based on the 6MWT values reported in a previous study (Ossowski et al., 2016) using G*Power software (version 3.1). A t-test was used to explore the difference between two independent means (two-tailed) with an effect size of Cohen’s d = 1.07, an alpha of 0.05, and a desired statistical power of 0.90. Based on these parameters, a total sample size of 40 participants (20 per group) was required. To account for potential dropouts or missing data, an additional 15% of participants were recruited beyond the calculated sample size.

Baseline demographic and anthropometric characteristics of the participants are presented in **Table 1**. Baseline RBC indices and physical performance parameters assessed before the intervention are presented in **Table 2**.

**Table 1:** Baseline characteristics of the study population (n=51).

Characteristics	BPT Group (n = 27)	DA group (n = 24)	*p*
Age (years)	69 ± 6	70 ± 3	0.823
Height (cm)	158 ± 6	160 ± 7	0.265
BW (kg)	69.3 ± 10.0	71.8 ± 13.0	0.440
BMI	27.6 ± 3.3	28.0 ± 4.9	0.750
RAC (cm)	32.3 ± 2.7	32.5 ± 3.9	0.787
LAC (cm)	32.2 ± 2.8	32.4 ± 3.9	0.824
WC (cm)	92.5 ± 10.0	95.8 ± 14.0	0.340
HC (cm)	98.8 ± 4.9	98.9 ± 6.6	0.993
WHR	0.93 ± 0.06	0.96 ± 0.08	0.139
RTC (cm)	52.4 ± 2.8	51.5 ± 3.5	0.327
LTC (cm)	52.6 ± 2.9	51.8 ± 3.5	0.338

BPT, Bungy Pump Training; DA, Daily Activity; BW, body weight; BMI, body-mass index; RAC, right arm circumference; LAC, left arm circumference; WC, waist circumference; HC, hip circumference; WHR, waist-hip ratio; RTC, right thigh circumference; LTC, left thigh circumference.

**Table 2:** Red blood cell indices, physical performance, and circumference before and after intervention in postmenopausal women (n = 51).

Outcome measure	BPT Group (n = 27)			DA group (n = 24)			Within-group comparison		Two-way ANOVA		
	Baseline	12 weeks	Change	Baseline	12 weeks	Change	BPT	DA	Time	Group	Interaction
							*P* (Cohen’s d)		*p* (η^2^)		
RBC (×10^9^L)	4.6 ± 0.3	4.5 ± 0.3 *	-0.1 ± 0.2	4.6 ± 0.3	4.5 ± 0.3	-0.1 ± 0.2	**0.001 (-0.33)**	0.204 (-0.33)	**0.002 (0.183)**	0.825 (0.001)	0.217 (0.031)
Hemoglobin (g/dl)	14.1 ± 0.7	13.7 ± 0.5 *	-0.5 ± 0.7	13.9 ± 0.9	13.6 ± 0.8	-0.2 ± 0.7	**0.001 (-0.66)**	0.158 (-0.35)	**0.001 (0.197)**	0.433 (0.013)	0.217 (0.031)
Hematocrit (%)	41.6 ± 1.9	41.0 ± 2.1	-0.5 ± 1.9	42.2 ± 2.7	41.4 ± 1.8	-0.8 ± 2.0	0.181 (-0.30)	0.060 (-0.35)	**0.020 (0.105)**	0.360 (0.017)	0.572 (0.007)
RDW-CV (%)	13.0 ± 0.6	12.9 ± 0.5	-0.1 ± 0.4	13.1 ± 0.7	13.2 ± 0.6 ^#^	0.1 ± 0.5	0.063 (-0.18)	0.393 (0.15)	0.751 (0.002)	0.181 (0.036)	0.075 (0.063)
MCV (fL)	90.1 ± 2.5	91.7 ± 2.5 *	1.6 ± 1.5	91.5 ± 3.3	90.2 ± 3.6 *	-1.3 ± 2.7	**0.001 (0.64)**	**0.022 (-0.38)**	0.618 (0.005)	0.960 (0.001)	**0.001 (0.332)**
MCH (pg)	30.5 ± 1.0	30.5 ± 1.0	0.0 ± 0.9	30.4 ± 1.2	29.9 ± 1.3 *	-0.5 ± 1.0	0.978 -	**0.031 (-0.40)**	0.227 (0.012)	0.108 (0.025)	0.269 (0.012)
CK (u/l)	99.6 ± 43.7	111.6 ± 30.8	12.0 ± 36.6	85.9 ± 32.0	92.1 ± 40.2	6.2 ± 33.9	0.100 (0.32)	0.376 (0.17)	0.071 (0.065)	0.076 (0.063)	0.562 (0.007)
6MWT (m)	656.1 ± 55.7	679.8 ± 58.3 *	23.7 ± 48.6	626.2 ± 52.2	644.6 ± 52.1 *^#^	18.5 ± 28.3	**0.018 (0.42)**	**0.004 (0.35)**	**0.001 (0.220)**	**0.027 (0.096)**	0.646 (0.004)
RAC (cm)	32.3 ± 2.7	31.7 ± 2.5 *	-0.6 ± 1.2	32.5 ± 3.9	32.3 ± 3.4	-0.2 ± 1.7	**0.019 (-0.23)**	0.536 (-0.05)	0.060 (0.070)	0.625 (0.005)	0.403 (0.014)
LAC (cm)	32.2 ± 2.8	31.6 ± 2.6 *	-0.7 ± 1.0	32.4 ± 3.9	32.2 ± 3.4	-0.2 ± 1.7	**0.002 (-0.26)**	0.587 (-0.05)	**0.032 (0.090)**	0.610 (0.005)	0.229 (0.029)
WC (cm)	92.5 ± 10.0	88.7 ± 9.6 *	-3.8 ± 3.4	95.8 ± 14.0	93.6 ± 12.6	-2.2 ± 5.5	**0.001 (-0.39)**	0.067 (-0.17)	**0.001 (0.314)**	0.206 (0.032)	0.196 (0.034)
HC (cm)	98.8 ± 4.9	98.1 ± 4.4	-0.7 ± 1.9	98.9 ± 6.6	99.4 ± 6.3	0.6 ± 2.8	0.060 (-0.15)	0.313 (0.10)	0.838 (0.001)	0.663 (0.004)	0.054 (0.074)
WHR	0.93 ± 0.06	0.90 ± 0.06 *	-0.03 ± 0.02	0.96 ± 0.08	0.94 ± 0.08 *	-0.02 ± 0.04	**0.001 (-0.5)**	**0.002 (-0.25)**	**0.001 (0.483)**	0.093 (0.057)	0.520 (0.008)
RTC (cm)	52.4 ± 2.8	52.4 ± 2.5	0.1 ± 1.5	51.5 ± 3.5	52.5 ± 3.6 *	1.0 ± 1.7	0.875 (0.04)	**0.010 (0.29)**	**0.028 (0.095)**	0.631 (0.005)	**0.044 (0.080)**
LTC (cm)	52.6 ± 2.9	52.7 ± 2.5	0.1 ± 1.7	51.8 ± 3.5	52.7 ± 3.7 *	1.0 ± 1.6	0.846 (0.04)	**0.009 (0.28)**	**0.035 (0.087)**	0.617 (0.005)	0.064 (0.068)

BPT, Bungy Pump Training; DA, Daily Activity; RBC, red blood cell; RDW-CV, red cell distribution width - coefficient of variation; MCV, mean corpuscular volume; MCH, mean corpuscular hemoglobin; CK, creatine kinase; 6MWT, 6-min walk test; RAC, right arm circumference; LAC, left arm circumference; WC, waist circumference; HC, hip circumference; WHR, waist-hip ratio; RTC, right thigh circumference; LTC, left thigh circumference. Bold values indicate statistically significant results. * Significantly different from baseline (p≤0.05). # Significantly different from the BPT group (p≤0.05).

### 2.3 Allocation

Participant allocation was performed by the study’s researcher (ZO) using a geographic location–based quasi-randomization approach, with participants assigned to the BPT group or the DA group in a simple 1:1 ratio. During the allocation process, two participants initially assigned to the DA group requested reassignment prior to the start of the intervention. These participants were reassigned before baseline intervention exposure, and the final group sizes remained consistent with the predefined sample size requirements. All analyses were conducted based on the final group allocation.

### 2.4 Intervention

The training program was designed in accordance with the ACSM guidelines for promoting health in older adults and was conducted over a 12-week period (36 training sessions, three sessions) (Nelson et al., 2007). Participants in the BPT group used poles with an integrated resistance shock absorber having an elastic resistance of 4 kg (Slimline Bungy Pump, Sports Progress International AB, Sweden). All training sessions were supervised by a certified Bungy Pump instructor accredited by the Polish Nordic Walking Federation, supported by at least two trained assistants, to ensure safety and correct execution of BPT techniques.

Each training session lasted approximately 60 minutes and consisted of three components: warm-up, main training, and cool-down. The warm-up phase included multifunctional exercises performed with the Bungy Pump poles to enhance the flexibility of the upper and lower limbs and trunk, as well as static and dynamic balance. The main training phase lasted approximately 40 minutes and consisted of three 10-minute bouts of marching interspersed with two 5-minute bouts of strength and balance exercises. Marching speed was progressively increased throughout the intervention, with participants covering approximately 1 km per 10 minutes. Strength and balance exercises were performed using the elastic resistance of the Bungy Pump poles, with 8–12 repetitions per movement. The 10-minute cool-down phase comprised static and dynamic flexibility exercises using the Bungy Pump poles.

Training sessions were conducted outdoors on an urban hill, with participants walking along designated paths. Exercise intensity was prescribed at 75–85% of age-predicted maximal heart rate (HRmax), calculated using the formula HRmax = 206 − 0.88 × age (Mansur and Nunes, 2010). Heart rate was continuously monitored during all sessions using Polar V800 heart rate monitors (Polar Electro Oy, Finland). Across the intervention period, participants progressively increased the total distance walked from approximately 2.8 to 3.6 km per session, achieving a mean heart rate of 114 ± 14 bpm (79% HRmax), consistent with moderate-to-vigorous to vigorous exercise intensity.

Participants assigned to the DA group served as a control condition and were instructed to maintain their habitual daily activities without participating in any structured or supervised exercise training throughout the study period. For ethical and health-related reasons, engagement in routine age-appropriate physical activities was permitted; however, participants were instructed to refrain from initiating new exercise programs. Physical activity levels were monitored for seven consecutive days using Polar V800 heart rate monitors (Polar Electro Oy, Finland), and participants were required to report their daily activities.

### 2.5 Outcome measures

All outcome measurements were conducted by trained assessors using standardized and validated procedures to ensure consistency and minimize measurement bias. Participants were instructed to maintain their habitual diet throughout the study period and to refrain from making significant changes to their nutritional intake. No formal dietary monitoring was conducted.

#### 2.5.1 Primary outcomes

##### 2.5.1.1 Hematological indices

Venous blood samples were collected in the morning (07:00–09:30) after a 12-hour overnight fast into EDTA-K_2_ tubes (Sarstedt, Germany). Participants were not instructed to restrict fluid intake beyond the fasting requirement; however, hydration status was not specifically assessed before blood collection. All analyses were conducted at an accredited clinical laboratory (Synevo Laboratory, Gdańsk, Poland) using the Sysmex XN-9100 system (XN-10 module), in accordance with the manufacturer’s diagnostic protocol (Sysmex Procedure A, 2019-08).

RBC and hematocrit were determined by direct current impedance. Hemoglobin was measured spectrophotometrically using the sodium lauryl sulfate hemoglobin method. Mean corpuscular volume (MCV), mean corpuscular hemoglobin (MCH), and RDW-CV were automatically calculated from primary indices. Fluorescence flow cytometry was applied for additional characterization of blood cell populations. Creatine kinase (CK) activity was determined spectrophotometrically by measuring light absorption at specific wavelengths.

Quality assurance procedures included the use of internal quality controls (Sysmex reference materials) and participation in external quality assessment schemes (RIQAS). All samples were analyzed using the same analyzer, and blood collections were performed at the same time points both before and after the intervention to minimize analytical and circadian variability.

##### 2.5.1.2 Physical performance

Physical performance was assessed using the 6MWT. The test was conducted on a 100-m athletic track marked at 10-m intervals. After a standardized warm-up, participants were instructed to walk continuously for six minutes at a self-selected pace. Time was recorded using a stopwatch, and heart rate was monitored throughout the test using a Polar V800 heart rate monitor (Polar Electro Oy, Kempele, Finland). The total distance covered during the six-minute period was recorded in meters (Ossowski et al., 2016).

#### 2.5.2 Secondary outcomes

Secondary outcomes included anthropometric measurements. Participants wore light clothing without metal components. Body weight (BW) was measured using a bioimpedance analyzer (InBody 720, Biospace, Seoul, Korea), and body height was measured with participants standing barefoot in an upright position. Circumference measurements were obtained using a non-elastic tape measure (GPM, Steckborn, Switzerland) with an accuracy of 0.1 cm (Liu et al., 2024; Measurement Toolkit - Simple measures - circumference, n.d). The tape was positioned horizontally and applied without compressing the skin. Arm circumference was measured at the midpoint between the acromion and the point of the olecranon process. WC was measured horizontally around the abdomen at the highest point of the iliac crest and the midpoint of the line connecting the lower border of the 12th rib. Hip circumference (HC) was measured at the widest part of the hip, above the trochanter (bony protuberance on the side of the femur). Thigh circumference was measured perpendicular to the long axis of the thigh at the midpoint between inguinal increase and the proximal border of the patella.

### 2.6 Statistical analysis

All statistical analyses and figure preparation were performed using SPSS version 24.0 (IBM Corp., Chicago, IL, USA) and GraphPad Prism version 9 (GraphPad Software, San Diego, CA, USA). Data were initially screened for potential outliers using Grubbs’ test. Missing outcome data were handled using multiple imputation. The normality of data distribution was assessed using histograms, Q–Q plots, and the Shapiro–Wilk test, while homogeneity of variances was examined using Levene’s test. A 2 × 2 repeated-measures ANOVA was used to examine the main effects of group, time and their interaction. For outcomes without significant omnibus effects, planned pairwise comparisons were conducted to examine within-group changes and between-group differences in pre-specified outcomes based on *a priori* clinical relevance. For non-normally distributed data, the Wilcoxon signed-rank test was used for within-group comparisons, and the Mann–Whitney U test was applied for between-group comparisons. Effect sizes for ANOVA outcomes were calculated using the partial eta squared (η^2^), with values of 0.02, 0.13 and 0.26 and above considered small, medium and large effect sizes (Boutcher et al., 2019). Effect sizes (Cohen’s d) were calculated to assess the magnitude of within-group changes and interpreted as small (0.2), moderate (0.5), and large (0.8) (Cohen, 1988). Data were expressed as mean ± standard deviation (SD) or median with interquartile range (IQR), where appropriate. Statistical significance was set at p < 0.05 for all analyses.

## 3 Results

### 3.1 Participant flow

**Figure 2:**
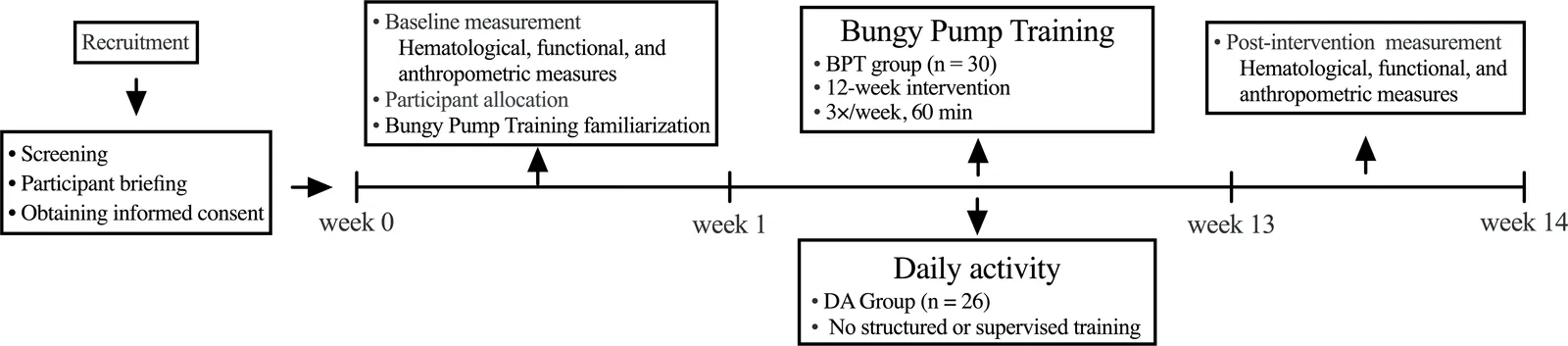
Flow chart of the study.

The flow of participants through the study is illustrated in **Figure 2**. Of the 121 individuals who initially expressed interest, 56 PW met the inclusion criteria and were enrolled in the study, with 30 allocated to the BPT group and 26 to the DA group. During the intervention period, three participants in the BPT group and two in the DA group withdrew for personal or other non–study-related reasons. Consequently, 27 participants (90.0%) in the BPT group and 24 participants (92.3%) in the DA group completed the post-intervention assessments.

### 3.2 Primary outcome

A significant group × time effect (interaction) was observed for MCV (p = 0.001, η^2^ = 0.332), indicating differential changes between groups. *Post hoc* tests showed a significant increase in MCV in the BPT group (p = 0.001, d = 0.64, 1.82 ± 1.71%) and a significant decrease in the DA group (p = 0.022, d = -0.38, −1.42 ± 2.88%).

Significant time effects were observed for RBC (p = 0.002, η^2^ = 0.183), hemoglobin (p = 0.001, η^2^ = 0.197), Hematocrit (p = 0.020, η^2^ = 0.105) and 6MWT (p = 0.001, η^2^ = 0.220). Following the intervention, the BPT group exhibited significant reductions in RBC (p = 0.001, d = -0.33, −2.9 ± 4.31%) and hemoglobin (p = 0.001, d = -0.66, −3.17 ± 4.72%), alongside a significant increase in 6MWT distance (p = 0.018, d = 0.42, 3.87 ± 7.62%). The DA group also demonstrated a significant improvement in 6MWT performance (p = 0.004, d = 0.35, 3.07 ± 4.66%), whereas no significant within-group changes in hematocrit were observed.

A significant group effect was observed for 6MWT (p = 0.027, η^2^ = 0.096), with a greater improvement in the BPT group compared with the DA group (p = 0.028, 0.81 ± 1.75%).

Beyond the ANOVA results, paired t-tests revealed a significant decrease in MCH (p = 0.031, d = -0.40, −1.56 ± 3.30%) in the DA group, and a significantly greater reduction in RDW-CV (p = 0.038, −1.84 ± 0.98%) in the BPT group compared with the DA group (**Table 2**).

### 3.3 Secondary outcomes

A significant interaction was observed for right thigh circumference (RTC, p = 0.044, η^2^ = 0.080), indicating differential changes between groups. *Post hoc* tests showed a significant increase in RTC in the DA group (p = 0.010, d = 0.29, 1.95 ± 3.44%), whereas no significant change was observed in the BPT group.

Significant time effects were observed for left arm circumference (LAC, p = 0.032, η^2^ = 0.090), WC (p = 0.001, η^2^ = 0.314), WHR (p = 0.001, η^2^ = 0.483), RTC (p = 0.028, η^2^ = 0.095), and left thigh circumference (LTC, p = 0.035, η^2^ = 0.087). Following the intervention, the BPT group exhibited significant reductions in LAC (p = 0.002, d = -0.26, −2 ± 3.02%), WC (p = 0.001, d = -0.39, −4.11 ± 3.50%) and WHR (p = 0.001, d = -0.5, −3.45 ± 2.63%). In contrast, the DA group showed significant increases in RTC (p = 0.01, d = 0.29, 1.95 ± 3.44%) and LTC (p = 0.009, d = 0.28, 1.87 ± 3.23%), along with a significant decrease in WHR (p = 0.002, d = -0.25, −2.65 ± 3.64%).

In addition, planned within-group comparisons revealed a significant reduction in right arm circumference (RAC, p = 0.019, d = -0.23, −1.65 ± 3.47%) in the BPT group (**Table 2**).

### 3.4 Safety and acceptability

No adverse events were recorded in participants during the study. The BPT group comprised 27 participants and completed a total of 36 training sessions. Across all intervention sessions, 97 absences were recorded, corresponding to an overall adherence rate of 90.0%. Attendance records showed an average participation rate at a satisfactory level.

## 4 Discussion

The present study examined the effects of a 12-week BPT program on RBC indices, physical performance, and anthropometric parameters in PW. The main findings were that BPT was associated with changes in hematological parameters, as indicated by alterations in RBC indices, alongside reductions in RBC and hemoglobin and an interaction in MCV. In parallel, improvements in 6MWT performance were observed, with greater increases in the BPT group compared with the DA group. Additionally, changes in anthropometric indicators were observed in the BPT group. Importantly, CK results suggested that the training protocol was well tolerated and did not induce excessive muscle damage.

CRF exhibits a strong inverse dose–response relationship with all-cause and CVD–related mortality (Han et al., 2022). The 6MWT is a widely used functional indicator of CRF, as it predicts VO_2_max and reflects the integrated responses of the cardiorespiratory, muscular, metabolic, and circulatory systems (ATS Committee on Proficiency Standards for Clinical Pulmonary Function Laboratories, 2002; Ross et al., 2010). In the present study, 6MWT distance significantly increased following the intervention, with a greater improvement observed in the BPT group compared with the DA group. Grigoletto et al. also showed a significant increase in 6MWT after 12 weeks of NW in middle-aged women (Grigoletto et al., 2022). Even modest increases in 6MWT distance are clinically meaningful in PW, as they are associated with improved functional capacity and reduced cardiovascular risk (Carter et al., 2024). Although a modest improvement was also observed in the DA group, the greater increase in the BPT group suggests that structured CT provides additional benefits beyond habitual activity. Collectively, these findings suggest that BPT may be a promising strategy for improving CRF in this population.

Hemoglobin within RBC plays a central role in oxygen transport and is a key determinant of aerobic capacity and VO_2_max (Bassett and Howley, 2000; Sitina et al., 2024). This is particularly relevant in PW, as menopause-related estrogen decline increases blood viscosity and impairs vascular and RBC function, potentially compromising microvascular perfusion and oxygen delivery (Sun et al., 2024; Mou et al., 2025). Thus, even modest exercise-induced changes in RBC indices may carry important physiological and clinical significance in this population. In the present study, BPT was associated with changes in RBC indices, including reductions in RBC and hemoglobin, along with an interaction in MCV. By increasing external resistance during locomotion, BPT enables simultaneous stimulation of the cardiovascular and musculoskeletal systems, which may contribute to exercise-induced physiological adaptations, consistent with the principles of combined training (Domaszewska et al., 2020; Marciniak et al., 2020). Previous studies have similarly reported improvements in cardiorespiratory efficiency, aerobic capacity, and functional fitness following BPT in PW, alongside adaptive changes in RBC indices (Domaszewska et al., 2020; Marciniak et al., 2020; Sobczak et al., 2023a). These hematological adaptations are considered physiological responses to training and are commonly attributed to plasma volume expansion rather than impaired erythropoiesis (Montero and Lundby, 2019). Importantly, hematocrit did not change significantly in either group, suggesting preserved red cell volume balance and supporting the interpretation of a physiological adaptation rather than a pathological response (Montero and Lundby, 2019; Sobczak et al., 2023a).

The coexistence of reduced RBC indices and hemoglobin concentration, together with improved 6MWT performance, may be consistent with exercise-induced hemodilution (often referred to as “sports anemia”), as described in previous studies (Damian et al., 2021). However, this interpretation remains speculative, as no direct measures of plasma volume or hemoglobin mass were obtained to distinguish between hemodilution and true reductions in red cell mass. Previous work by Sobczak et al. reported reductions in RBC and hematocrit following eight weeks of twice-weekly BPT in PW (Sobczak et al., 2023a). The more pronounced hemoglobin reduction observed in the present study may be related to the longer intervention duration and the higher training intensity (~79% HRmax), both of which are known to influence hematological adaptations to exercise.

In addition to these adaptations, RDW has emerged as an independent prognostic biomarker for CVDs, with elevated RDW reflecting impaired RBC deformability, increased inflammation, and altered vascular flow (Sobczak et al., 2023b). NW has been suggested to reduce RDW by limiting RBC size heterogeneity, potentially through enhanced erythropoiesis, reduced oxidative stress, and attenuation of systemic inflammation (Danese et al., 2015; Gomarasca et al., 2022). In the present study, a statistically significant between-group difference in RDW-CV change was observed, with a greater reduction in the BPT group compared with the DA group. Previous studies have reported a decrease in RDW-CV following BPT in PW (Sobczak et al., 2023a). However, in contrast to these findings, within-group changes in RDW-CV were not statistically significant in either group in the present study. Therefore, this result should be interpreted with caution, as the observed between-group difference may partly reflect changes in the DA group rather than a definitive effect of BPT. To date, evidence regarding the effects of NW, including BPT, on RDW-related parameters remains limited, and RDW-CV is rarely included as an outcome measure in such studies. Therefore, the present findings contribute to a relatively underexplored area of research but should be considered preliminary.

A reduction in RDW-CV may reflect improved RBC homeostasis and deformability, which may lower blood viscosity and enhance microvascular perfusion, thereby contributing to reduced cardiovascular risk (Salvagno et al., 2015). In addition, reductions in RDW-CV may be partially mediated by improvements in systemic inflammation and oxidative stress, both of which are known to influence RBC heterogeneity (Arkew et al., 2022). Furthermore, reductions in RDW-CV have been associated with improvements in aerobic capacity; notably, Sobczak et al. observed a concurrent decrease in RDW-CV and increase in VO_2_max following BPT (Sobczak et al., 2023a). In a related study conducted in the same population, a significant within-group improvement in VO_2max_ was observed in the BPT group, suggesting that BPT may induce meaningful cardiorespiratory adaptations (Niu et al., 2025). In the present study, a similar directional pattern between RDW-CV change and improvement in 6MWT performance was observed; however, given the absence of significant within-group changes in RDW-CV, this observation should be interpreted with caution. While these findings may indicate a potential link between hematological and functional adaptations, they do not provide definitive evidence that BPT improves RDW-CV. Overall, the findings should be interpreted as associations rather than direct evidence of underlying physiological mechanisms, given the absence of direct mechanistic measurements. Further studies with larger sample sizes and more rigorous designs are warranted to clarify this relationship.

WC and WHR are well-established indicators of central adiposity and are strongly associated with cardiovascular and metabolic risk (Czernichow et al., 2011; Gordito Soler et al., 2025). In the present study, BPT was associated with reductions in WC and WHR, findings that are consistent with previous reports in PW and further support the beneficial effects of structured exercise on visceral fat accumulation (Domaszewska et al., 2020; Sobczak et al., 2023a). These changes are particularly relevant in PW, in whom menopause-related hormonal alterations promote central obesity and insulin resistance, thereby substantially increasing cardiovascular risk (El Khoudary et al., 2020; Marsh et al., 2023). Notably, Sobczak et al. previously reported a significant correlation between RDW and WC in PW (Sobczak et al., 2023a), suggesting a link between central adiposity and hematological status. Reductions in WC and WHR may reflect changes in body composition and central adiposity; however, direct assessment of fat mass was not performed in the present study. Therefore, these findings may be associated with favorable physiological and functional adaptations.

In addition to central adiposity, reductions in upper arm circumference were observed in the BPT group. These changes may reflect decreases in subcutaneous fat rather than losses in functional muscle mass, particularly given the concurrent improvements in 6MWT performance and the established functional benefits of NW (Frisancho, 1981; Mathieson and Lin, 2014). Previous studies indicate that NW–based interventions increase upper-limb muscle activation and overall metabolic demand, but do not consistently induce hypertrophic adaptations of the upper limbs, suggesting that pole-assisted locomotion predominantly elicits metabolic and functional responses rather than muscle mass–oriented adaptations (Mathieson and Lin, 2014; Pellegrini et al., 2015). Collectively, the anthropometric adaptations observed in this study provide important metabolic context for the hematological and functional improvements induced by BPT and further support its potential role as a non-pharmacological strategy for CVDs prevention in PW.

Given its growing popularity and ease of implementation, BPT may be incorporated into community-based exercise programs targeting healthy aging. Future studies should examine different training intensities, longer intervention durations, and comparative effectiveness against other exercise modalities.

The present study provides evidence that a 12-week BPT program is associated with changes in RBC indices and improvements in physical performance in PW. All participants completed the intervention without adverse events, indicating that BPT is a safe and well-tolerated exercise modality in this population. The observed changes in RBC indices, together with improvements in 6MWT performance, suggest that BPT may promote integrated hematological and functional adaptations. Although favorable changes in anthropometric parameters were also observed, these findings should be interpreted as secondary outcomes. Overall, BPT may represent a feasible non-pharmacological strategy to support functional capacity and aspects of cardiovascular health in PW. Given its growing popularity and ease of implementation, BPT may be incorporated into community-based exercise programs targeting healthy aging. Future studies should examine different training intensities, longer intervention durations, and comparative effectiveness against other exercise modalities.

This study has several strengths: (1) all training sessions were supervised by a certified Bungy Pump instructor, ensuring standardized exercise delivery and participant safety. (2) outcome assessments were performed by blinded assessors, reducing the risk of measurement bias. (3) The intervention combined aerobic and resistance components within a single training modality, aligning with current exercise recommendations for older adults. (4) participants were recruited from diverse community settings, which may enhance the generalizability of the findings. (5) The study included a comprehensive evaluation of both hematological parameters, including RBC indices, and functional outcomes, providing an integrated assessment of physiological adaptations to BPT.

Several limitations should be acknowledged. (1) Due to the nature of the intervention, blinding of the exercise instructor was not feasible; however, assessor blinding was implemented to minimize potential bias. (2) Although the final group sizes were not perfectly balanced, each group met the predefined sample size requirements. (3) Importantly, although a between-group difference in RDW-CV was observed, the absence of significant within-group changes limits the interpretation of this finding, and RDW-CV should be considered an exploratory outcome in the present study. In addition, hydration status was not specifically assessed prior to blood sampling, which may have influenced hematological parameters. (4) The intervention was conducted under supervised conditions, which may limit the generalizability of the findings to unsupervised or real-world settings. Future studies should evaluate the effectiveness of BPT under less controlled conditions and in larger populations. (5) There was a lack of a detailed dietary assessment. Although participants were instructed to maintain their habitual diet, no formal monitoring was conducted. Therefore, potential variations in dietary intake cannot be excluded and may have influenced the results. Future studies should incorporate validated dietary assessment methods.

## 5 Conclusion

This study provides evidence that a 12-week BPT program is associated with changes in RBC indices and improvements in physical performance in PW. BPT was safe and well-tolerated, and was associated with improvements in functional capacity and selected anthropometric parameters. While these findings suggest that BPT may represent a feasible non-pharmacological strategy to support CRF and aspects of cardiovascular health, further studies are required to confirm these effects.

## Data Availability

The raw data supporting the conclusions of this article will be made available by the authors, without undue reservation.

## References

[B1] AlGhatrif M. Morrell C. H. Fleg J. L. Chantler P. D. Najjar S. S. Becker L. C. . (2024). Longitudinal decline in peak V̇o2 with aging in a healthy population is associated with a reduction in peripheral oxygen utilization but not in cardiac output. Am. J. Physiol. Heart Circ. Physiol. 327, H509–H517. doi: 10.1152/ajpheart.00665.2023 38874616 PMC11442097

[B2] Arkew M. Gemechu K. Haile K. Asmerom H. (2022). Red blood cell distribution width as novel biomarker in cardiovascular diseases: a literature review. J. Blood Med. 13, 413–424. doi: 10.2147/JBM.S367660 35942475 PMC9356613

[B3] ATS Committee on Proficiency Standards for Clinical Pulmonary Function Laboratories (2002). ATS statement: guidelines for the six-minute walk test. Am. J. Respir. Crit. Care Med. 166, 111–117. doi: 10.1164/ajrccm.166.1.at1102 12091180

[B4] Measurement Toolkit - Simple measures - circumference. Available online at: https://www.measurement-toolkit.org/anthropometry/objective-methods/simple-measures-circumference (Accessed March 3, 2026).

[B5] Bassett D. R. Howley E. T. (2000). Limiting factors for maximum oxygen uptake and determinants of endurance performance. Med. Sci. Sports Exerc 32, 70–84. doi: 10.1097/00005768-200001000-00012 10647532

[B6] Boreskie K. F. Rose A. V. Hay J. L. Kehler D. S. Costa E. C. Moffatt T. L. . (2020). Frailty status and cardiovascular disease risk profile in middle-aged and older females. Exp. Gerontology 140, 111061. doi: 10.1016/j.exger.2020.111061 32814098

[B7] Boutcher Y. N. Boutcher S. H. Yoo H. Y. Meerkin J. D. (2019). The effect of sprint interval training on body composition of postmenopausal women. Med. Sci. Sports Exerc 51, 1413–1419. doi: 10.1249/MSS.0000000000001919 31210647

[B8] Carter S. J. Blechschmid T. H. Baranauskas M. N. Long E. B. Gruber A. H. Raglin J. S. . (2024). Preworkout dietary nitrate magnifies training-induced benefits to physical function in late postmenopausal women: a randomized pilot study. Am. J. Physiol. - Regul. Integr. Comp. Physiol. 327, R534–R542. doi: 10.1152/ajpregu.00150.2024 39250543 PMC11687826

[B9] Cohen J. (1988). Statistical power analysis for the behavioral sciences (New York, NY: Psychology Press).

[B10] Czernichow S. Kengne A.-P. Stamatakis E. Hamer M. Batty G. D. (2011). Body mass index, waist circumference and waist-hip ratio: which is the better discriminator of cardiovascular disease mortality risk?: evidence from an individual-participant meta-analysis of 82–864 participants from nine cohort studies. Obes. Rev. 12, 680–687. doi: 10.1111/j.1467-789X.2011.00879.x 21521449 PMC4170776

[B11] Damian M.-T. Vulturar R. Login C. C. Damian L. Chis A. Bojan A. (2021). Anemia in sports: a narrative review. Life. (Basel) 11, 987. doi: 10.3390/life11090987 34575136 PMC8472039

[B12] Danese E. Lippi G. Montagnana M. (2015). Red blood cell distribution width and cardiovascular diseases. J. Thorac. Dis. 7, E402–E411. doi: 10.3978/j.issn.2072-1439.2015.10.04 26623117 PMC4635283

[B13] Dhamayanti A. S. Rahmad R. Rachmawati S. Waranugraha Y. (2025). A systematic review and meta-analysis of Nordic walking for chronic heart failure with low left ventricular ejection fraction. PM R. J. Inj. Funct. Rehabil. 17, 211–221. doi: 10.1002/pmrj.13254 39215750

[B14] Domaszewska K. Koper M. Wochna K. Czerniak U. Marciniak K. Wilski M. . (2020). The effects of Nordic walking with poles with an integrated resistance shock absorber on cognitive abilities and cardiopulmonary efficiency in postmenopausal women. Front. Aging Neurosci. 12. doi: 10.3389/fnagi.2020.586286 33192480 PMC7604469

[B15] El Khoudary S. R. Aggarwal B. Beckie T. M. Hodis H. N. Johnson A. E. Langer R. D. . (2020). Menopause transition and cardiovascular disease risk: implications for timing of early prevention: a scientific statement from the American Heart Association. Circulation 142, e506–e532. doi: 10.1161/CIR.0000000000000912 33251828

[B16] Ferreira L. Abrantes C. Alves M. E. Moreira C. Moreira H. (2024). Effects of exercise programs on cardiorespiratory fitness and arterial stiffness on postmenopausal women: a systematic review study. Maturitas 181, 107917. doi: 10.1016/j.maturitas.2024.107917 38277884

[B17] Frisancho A. R. (1981). New norms of upper limb fat and muscle areas for assessment of nutritional status. Am. J. Clin. Nutr. 34, 2540–2545. doi: 10.1093/ajcn/34.11.2540 6975564

[B18] Gifford J. R. Collins J. (2021). Critical speed throughout aging: insight into the world masters championships. Med. Sci. Sports Exercise 53, 524–533. doi: 10.1249/MSS.0000000000002501 33560767

[B19] Gomarasca M. Micielska K. Faraldi M. Flis M. Perego S. Banfi G. . (2022). Impact of 12-week moderate-intensity aerobic training on inflammasome complex activation in elderly women. Front. Physiol. 13. doi: 10.3389/fphys.2022.792859 35273516 PMC8902397

[B20] Gordito Soler M. Tárraga López P. J. López-González Á.A. Paublini H. Martínez-Almoyna Rifá E. Vicente-Herrero M. T. . (2025). Is measuring BMI and waist circumference as good in assessing insulin resistance as using bioelectrical impedance to measure total body fat and visceral fat? Diabetology 6, 32. doi: 10.3390/diabetology604003241725453

[B21] Grigoletto A. Mauro M. Oppio A. Greco G. Fischetti F. Cataldi S. . (2022). Effects of Nordic walking training on anthropometric, body composition and functional parameters in the middle-aged population. Int. J. Environ. Res. Public Health 19, 7433. doi: 10.3390/ijerph19127433 35742680 PMC9224194

[B22] Han M. Qie R. Shi X. Yang Y. Lu J. Hu F. . (2022). Cardiorespiratory fitness and mortality from all causes, cardiovascular disease and cancer: dose-response meta-analysis of cohort studies. Br. J. Sports Med. 56, 733–739. doi: 10.1136/bjsports-2021-104876 35022163

[B23] Haybar H. Pezeshki S. M. S. Saki N. (2019). Evaluation of complete blood count parameters in cardiovascular diseases: an early indicator of prognosis? Exp. Mol. Pathol. 110, 104267. doi: 10.1016/j.yexmp.2019.104267 31194963

[B24] Huta-Osiecka A. Wochna K. Stemplewski R. Marciniak K. Podgórski T. Kasprzak Z. . (2022). Influence of Nordic walking with poles with an integrated resistance shock absorber on carbohydrate and lipid metabolic indices and white blood cell subpopulations in postmenopausal women. PeerJ 10, e13643. doi: 10.7717/peerj.13643 35791365 PMC9250761

[B25] Janssen I. (2010). Evolution of sarcopenia research. Appl. Physiol. Nutr. Metab. 35, 707–712. doi: 10.1139/H10-067 20962927

[B26] Jiang M. Ren X. Han L. Zheng X. (2024). Associations between sarcopenic obesity and risk of cardiovascular disease: a population-based cohort study among middle-aged and older adults using the CHARLS. Clin. Nutr. 43, 796–802. doi: 10.1016/j.clnu.2024.02.002 38350287

[B27] Khalafi M. Sakhaei M. H. Habibi Maleki A. Rosenkranz S. K. Pourvaghar M. J. Fang Y. . (2023). Influence of exercise type and duration on cardiorespiratory fitness and muscular strength in post-menopausal women: a systematic review and meta-analysis. Front. Cardiovasc. Med. 10. doi: 10.3389/fcvm.2023.1190187 37229231 PMC10204927

[B28] Lang J. J. Prince S. A. Merucci K. Cadenas-Sanchez C. Chaput J.-P. Fraser B. J. . (2024). Cardiorespiratory fitness is a strong and consistent predictor of morbidity and mortality among adults: an overview of meta-analyses representing over 20.9 million observations from 199 unique cohort studies. Br. J. Sports Med. 58, 556–566. doi: 10.1136/bjsports-2023-107849 38599681 PMC11103301

[B29] Liu J. Kim J.-H. (2025). The effects of nordic walking on the cardiovascular risk factors in older adults: a systematic review and meta-analysis. Arch. Gerontol. Geriatr. 129, 105663. doi: 10.1016/j.archger.2024.105663 39476525

[B30] Liu Y. Mao S. Xie W. Agnieszka H.-L. K. Helena S. M. Magdalena D.-Z. . (2024). Relationship between physical activity and abdominal obesity and metabolic markers in postmenopausal women. Sci. Rep. 14, 26496. doi: 10.1038/s41598-024-77900-x 39489777 PMC11532536

[B31] Mansur A. J. Nunes R. A. B. (2010). Heart rate response and chronotropic incompetence in exercise stress testing of asymptomatic women. Womens Health (Lond) 6, 785–787. doi: 10.2217/whe.10.66 21118037

[B32] Mänttäri A. Suni J. Sievänen H. Husu P. Vähä-Ypyä H. Valkeinen H. . (2018). Six-minute walk test: a tool for predicting maximal aerobic power (VO2 max) in healthy adults. Clin. Physiol. Funct. Imaging. 38, 1038–1045. doi: 10.1111/cpf.12525 29851229

[B33] Marciniak K. Maciaszek J. Cyma-Wejchenig M. Szeklicki R. Maćkowiak Z. Sadowska D. . (2020). The effect of Nordic walking training with poles with an integrated resistance shock absorber on the functional fitness of women over the age of 60. Int. J. Environ. Res. Public Health 17, 2197. doi: 10.3390/ijerph17072197 32218296 PMC7177745

[B34] Marsh M. L. Oliveira M. N. Vieira-Potter V. J. (2023). Adipocyte metabolism and health after the menopause: the role of exercise. Nutrients 15, 444. doi: 10.3390/nu15020444 36678314 PMC9862030

[B35] Mathieson S. Lin C.-W. C. (2014). Health benefits of Nordic walking; a systematic review. Br. J. Sports Med. 48, 1577–1578. doi: 10.1136/bjsports-2013-093294 24505040

[B36] Montero D. Lundby C. (2019). Regulation of red blood cell volume with exercise training. Compr. Physiol. 9, 149–164. doi: 10.1002/j.2040-4603.2019.tb00060.x 30549016

[B37] Mou H. Zhang J. Guo Y. Xu L. Luo X. (2025). Effects of key physiological parameters on cardiovascular disease and osteoporosis risk in perimenopausal and postmenopausal women. Sci. Rep. 15, 2814. doi: 10.1038/s41598-025-86613-8 39843604 PMC11754902

[B38] Nebeck K. Gelaye B. Lemma S. Berhane Y. Bekele T. Khali A. . (2012). Hematological parameters and metabolic syndrome: findings from an occupational cohort in Ethiopia. Diabetes Metab. Syndrome: Clin. Res. Rev. 6, 22–27. doi: 10.1016/j.dsx.2012.05.009 23014250 PMC3460271

[B39] Nelson M. E. Rejeski W. J. Blair S. N. Duncan P. W. Judge J. O. King A. C. . (2007). Physical activity and public health in older adults: recommendation from the American College of Sports Medicine and the American Heart Association. Med. Sci. Sports Exerc 39, 1435–1445. doi: 10.1249/mss.0b013e3180616aa2 17762378

[B40] Niu Y. Radzimiński Ł. Qian G. Szwarc A. Ossowski Z. (2025). Effect of a 12-week bungy pump training on cardiorespiratory fitness, metabolic syndrome factors and body composition in postmenopausal women. Sci. Rep. 15, 41938. doi: 10.1038/s41598-025-25909-1 41290844 PMC12647649

[B41] Nunes P. R. P. Castro-e-Souza P. de Oliveira A. A. Camilo B. F. Cristina-Souza G. Vieira-Souza L. M. . (2024). Effect of resistance training volume on body adiposity, metabolic risk, and inflammation in postmenopausal and older females: systematic review and meta-analysis of randomized controlled trials. J. Sport Health Sci. 13, 145–159. doi: 10.1016/j.jshs.2023.09.012 37788790 PMC10980902

[B42] Nurrobi Y. A. S. Winston K. Rahman A. L. Falaki M. F. Aristya M. P. Toaha A. F. . (2024). Footsteps to wellness: a systematic review and meta-analysis of walking pace and coronary artery disease event. Cureus 16, e56926. doi: 10.7759/cureus.56926 38665701 PMC11043612

[B43] Ossowski Z. M. Skrobot W. Aschenbrenner P. Cesnaitiene V. J. Smaruj M. (2016). Effects of short-term Nordic walking training on sarcopenia-related parameters in women with low bone mass: a preliminary study. Clin. Interv Aging 11, 1763–1771. doi: 10.2147/CIA.S118995 27942207 PMC5137931

[B44] Paluch A. E. Bajpai S. Ballin M. Bassett D. R. Buford T. W. Carnethon M. R. . (2023). Prospective association of daily steps with cardiovascular disease: a harmonized meta-analysis. Circulation 147, 122–131. doi: 10.1161/CIRCULATIONAHA.122.061288 36537288 PMC9839547

[B45] Pellegrini B. Peyré-Tartaruga L. A. Zoppirolli C. Bortolan L. Bacchi E. Figard-Fabre H. . (2015). Exploring muscle activation during nordic walking: a comparison between conventional and uphill walking. PloS One 10, e0138906. doi: 10.1371/journal.pone.0138906 26418339 PMC4587792

[B46] Powell-Wiley T. M. Poirier C. P. Burke V. C. L. E. Després J.-P. Gordon-Larsen P. Lavie C. J. . (2021). Obesity and cardiovascular disease. Circulation 143, e984–e1010. doi: 10.1161/CIR.0000000000000973 33882682 PMC8493650

[B47] Rodziewicz-Flis E. Juhas U. Kortas J. A. Jaworska J. Bidzan-Bluma I. Babińska A. . (2023). Nordic walking training in BungyPump form improves cognitive functions and physical performance and induces changes in amino acids and kynurenine profiles in older adults. Front. Endocrinol. (Lausanne) 14. doi: 10.3389/fendo.2023.1151184 37766686 PMC10520281

[B48] Ross R. M. Murthy J. N. Wollak I. D. Jackson A. S. (2010). The six minute walk test accurately estimates mean peak oxygen uptake. BMC Pulm. Med. 10, 31. doi: 10.1186/1471-2466-10-31 20504351 PMC2882364

[B49] Ruiz-Rios M. Maldonado-Martin S. (2022). Physical activity on cardiorespiratory fitness and cardiovascular risk in premenopausal and postmenopausal women: a systematic review of randomized controlled trials. Menopause 29, 1222. doi: 10.1097/GME.0000000000002037 35969888

[B50] Salvagno G. L. Sanchis-Gomar F. Picanza A. Lippi G. (2015). Red blood cell distribution width: a simple parameter with multiple clinical applications. Crit. Rev. Clin. Lab. Sci. 52, 86–105. doi: 10.3109/10408363.2014.992064 25535770

[B51] Sanchis-Gomar F. Lavie C. J. Marín J. Perez-Quilis C. Eijsvogels T. M. H. O’Keefe J. H. . (2022). Exercise effects on cardiovascular disease: from basic aspects to clinical evidence. Cardiovasc. Res. 118, 2253–2266. doi: 10.1093/cvr/cvab272 34478520

[B52] Sitina M. Stark H. Schuster S. (2024). Optimal hematocrit theory: a review. J. Appl. Physiol. 137, 494–509. doi: 10.1152/japplphysiol.00034.2024 38813609

[B53] Skórkowska-Telichowska K. Kropielnicka K. Bulińska K. Pilch U. Wozniewski M. Szuba A. . (2016). Nordic walking in the second half of life. Aging Clin. Exp. Res. 28, 1035–1046. doi: 10.1007/s40520-016-0531-8 26803510

[B54] Sobczak K. Nowinka P. Wochna K. Domaszewska K. (2023a). The effects of nordic walking with poles with an integrated resistance shock absorber on red blood cell distribution and cardiorespiratory efficiency in postmenopausal women—a randomized controlled trial. Biol. (Basel) 12, 179. doi: 10.3390/biology12020179 36829458 PMC9952538

[B55] Sobczak K. Wochna K. Antosiak-Cyrak K. Domaszewska K. (2023b). The effects of 6-month aqua aerobics training on cardiometabolic parameters in perimenopausal women-a randomized controlled trial. Biol. (Basel) 12, 588. doi: 10.3390/biology12040588 37106789 PMC10136125

[B56] Srikanthan P. Karlamangla A. S. (2014). Muscle mass index as a predictor of longevity in older adults. Am. J. Med. 127, 547–553. doi: 10.1016/j.amjmed.2014.02.007 24561114 PMC4035379

[B57] Sun W. Han Y. Gu S. (2024). Effects of five types of exercise on vascular function in postmenopausal women: a network meta-analysis and systematic review of 32 randomized controlled trials. PeerJ 12, e17621. doi: 10.7717/peerj.17621 39026541 PMC11257064

[B58] Tsampasian V. Bloomfield G. S. (2025). The evolving global burden of cardiovascular diseases: what lies ahead. Eur. J. Prev. Cardiol. 32, 1016–1017. doi: 10.1093/eurjpc/zwae330 39365755

[B59] Vaduganathan M. Mensah G. A. Turco J. V. Fuster V. Roth G. A. (2022). The global burden of cardiovascular diseases and risk: a compass for future health. J. Am. Coll. Cardiol. 80, 2361–2371. doi: 10.1016/j.jacc.2022.11.005 36368511

[B60] Wiacek M. Natora J. Zubrzycki I. Z. Tomasiuk R. (2023). Physiological responses associated with nordic-walking and walking in middle-age women. Int. J. Sports Med. 44, 865–870. doi: 10.1055/a-2134-3769 37751766 PMC10622228

[B61] Wochna K. Ogurkowska M. Leszczyński P. Stemplewski R. Huta-Osiecka A. Błaszczyk A. . (2022). Nordic walking with an integrated resistance shock absorber affects the femur strength and muscles torques in postmenopausal women. Sci. Rep. 12, 20089. doi: 10.1038/s41598-022-24131-7 36418455 PMC9684118

[B62] Xia W. Khalil R. A. (2025). Hormone replacement therapy and cardiovascular health in postmenopausal women. Int. J. Mol. Sci. 26, 5078. doi: 10.3390/ijms26115078 40507889 PMC12154064

[B63] Yang K. Hou R. Zhao J. Wang X. Wei J. Pan X. (2023). Lifestyle effects on aging and CVD: a spotlight on the nutrient-sensing network. Ageing Res. Rev. 92, 102121. doi: 10.1016/j.arr.2023.102121 37944707

